# Marked attenuation of the amplitude of transcranial motor-evoked potentials after intravenous bolus administration of ketamine: a case report

**DOI:** 10.1186/s13256-018-1741-9

**Published:** 2018-07-13

**Authors:** Kenta Furutani, Mari Matsuhashi, Hiroyuki Deguchi, Yusuke Mitsuma, Nobuko Ohashi, Hiroshi Baba

**Affiliations:** 1 Department of Anesthesiology, Uonuma Institute of Community Medicine, Niigata University Medical and Dental Hospital, 4132 Urasa, Minami-Uonuma, Niigata, 949-7302 Japan; 2 Department of Anesthesiology, Niigata University Medical and Dental Sciences, 1-757 Asahimachi-Dori, Chuo-ku, Niigata, 951-8510 Japan

**Keywords:** Bolus administration, Ketamine, Monitoring, Motor-evoked potentials, Spine surgery

## Abstract

**Background:**

It is believed that ketamine does not affect motor-evoked potential amplitude, whereas various anesthetic drugs attenuate the amplitude of transcranial motor-evoked potential. However, we encountered a patient with marked attenuation of motor-evoked potential amplitude after intravenous bolus administration of ketamine.

**Case presentation:**

A 15-year-old Japanese girl with a diagnosis of adolescent idiopathic scoliosis was admitted to our hospital to undergo posterior spinal fusion at T4–L3. After induction of general anesthesia using a continuous infusion of propofol and remifentanil, we confirmed that transcranial electrical motor-evoked potentials were being recorded correctly. Ketamine 1.25 mg/kg was administered intravenously for intraoperative and postoperative analgesia. About 3 minutes later, the motor-evoked potential amplitude was markedly attenuated. No other drugs were administered except for ketamine. The patient’s vital signs were stable, and the surgery had not yet started. The motor-evoked potential amplitude was recovered at about 6 minutes after administration of ketamine. The surgery was performed uneventfully, and the patient had no neurologic deficit when she emerged from general anesthesia.

**Conclusions:**

Although there is a widely held belief in the field of anesthesiology that ketamine does not affect motor-evoked potential amplitude, it has been suggested that ketamine could affect its monitoring.

## Background

Various anesthetic drugs, including volatile anesthetic agents, propofol, and midazolam, attenuate the amplitude of transcranial motor-evoked potentials (MEPs) [1, 2]. It is believed that ketamine does not affect the MEP amplitude [3]. Further, ketamine has an analgesic effect as well as a hypnotic effect. Continuous infusion of ketamine combined with opioids is known to attenuate postoperative pain after complex spine surgery [4], which is considered to be a particularly painful procedure [5]. Therefore, ketamine is a useful drug in complex spine surgery for both recording MEPs and improving the quality of postoperative analgesia. However, although there are no reports of ketamine reducing the amplitude of MEPs, we encountered a case of marked attenuation of the MEP amplitude after intravenous bolus administration of ketamine before posterior spinal fusion in a patient with adolescent idiopathic scoliosis.

## Case presentation

A 15-year-old Japanese girl with a diagnosis of adolescent idiopathic scoliosis was admitted to our hospital to undergo posterior spinal fusion at T4–L3. She was a high school student and did not present any symptoms at admission. She did not take any medications prior to the surgery. Her past medical, social, environmental, and family history was not appreciable. She was 147 cm tall, and her weight was 40 kg. She had a Cobb angle of 60 degrees. She had no neurological symptoms. Her temperature was 36.8 °C, her blood pressure was 118/64 mmHg, and her pulse was 92 beats per minute. Laboratory findings at admission were as follows. Her white blood cell count was 6860/μl, red blood cell count 472 × 10^4^/μl, hemoglobin 14.3 g/dl, hematocrit 42.1%, platelets 30.8 × 10^4^/μl, aspartate transaminase 19 IU/L, alanine transaminase 15 IU/L, total bilirubin 0.5 mg/dl, γ-glutamyl transferase 16 IU/L, alkaline phosphatase 371 IU/L, total protein 8.2 g/dl, albumin 5.2 g/dl, blood urea nitrogen 11 mg/dl, creatinine 0.39 mg/dl, sodium 141 mEq/L, potassium 4.5 mEq/L, chloride 102 mEq/L, C-reactive protein 0.01 mg/dl, urinalysis pH 6.0, no uric protein, no urinary sugar, no ketone body, and no uric blood. No microbial examination was performed.

We planned general anesthesia using a target-controlled infusion of propofol and a continuous infusion of remifentanil to record MEPs and somatosensory evoked potentials (SSEPs). In addition, we planned to use both bolus and continuous infusions of ketamine, followed by intravenous patient-controlled fentanyl as postoperative analgesia because we believed that ketamine did not affect MEP monitoring.

After securing an intravenous line in the patient’s forearm, general anesthesia was induced using a target-controlled infusion of propofol 4 μg/ml and remifentanil 0.3 μg/kg/minute. After administration of rocuronium 20 mg, the trachea was intubated using a reinforced endotracheal tube. Thereafter, 1 g of cefazolin sodium was administered intravenously every 3 hours during the surgery and every 12 hours until postoperative day 1.

We started preparation for recording the MEPs and SSEPs. MEPs were evoked by transcranial electrical stimulation (a train of five pulses with an interstimulus interval of 2 milliseconds; supramaximal stimulus, 400 V) with screw electrodes fixed at 2 cm anterior to C3 and C4 (cathode and anode, respectively, following the international 10–20 system). MEPs and SSEPs were recorded using an intraoperative neurophysiologic monitoring system (Neuromaster MEE-1216; Nihon Kohden, Tokyo, Japan). MEPs were recorded bilaterally from the abductor pollicis, quadriceps femoris, tibialis anterior, and flexor hallucis brevis muscles. SSEPs were evoked by stimulation (30 mA) of the sciatic nerve and recorded from 2 cm posterior to Cz (averaging the signals generated by 200 stimulations).

**Fig. 1:**
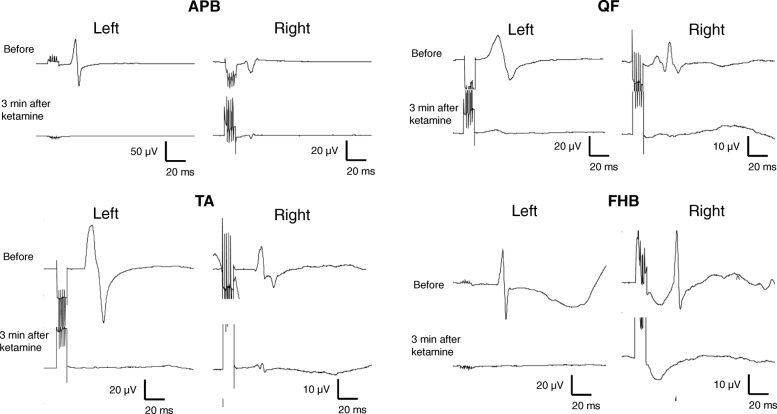
Traces showing the motor-evoked potentials in our patient. Three minutes after administration of ketamine, there was a significant decrease in the amplitudes of motor-evoked potentials recorded from eight muscles. *APB* Abductor pollicis muscle, *QF* Quadriceps femoris muscle, *TA* Tibialis anterior muscle, *FHB* Flexor hallucis brevis muscle

After the patient was turned to a prone position, we confirmed that we could record MEPs from all targeted muscles without difficulty. We maintained general anesthesia using a target-controlled infusion of propofol 3.3 μg/ml and remifentanil 0.2 μg/kg/minute. The patient’s bispectral index values were below 60, and her vital signs were stable. Several minutes before the skin incision was made, the attending anesthesiologist evaluating the MEPs discovered that they were markedly attenuated (Fig. 1). The attending anesthesiologist had not changed the propofol infusion rate and had not administered any other drugs except for intravenous bolus administration of ketamine 50 mg (1.25 mg/kg). Ketamine was administered about 3 minutes before the change in MEP waveforms. The patient’s blood pressure, heart rate, peripheral oxygen saturation, end-tidal CO_2_, and body temperature remained stable. The surgery had not started at that time. About 6 minutes after administration of ketamine, the MEP amplitudes were recovered.

The surgery was performed uneventfully, and the patient had no neurologic deficit when she emerged from general anesthesia. Intravenous patient-controlled analgesia (a combination of fentanyl 25 μg/ml and ketamine 2.5 mg/ml, background infusion 1 ml/hour, bolus 1 ml, and lockout time 10 minutes) was used for postoperative pain management. The patient was discharged on postoperative day 11 without any complications. She was well without complications at 2 years after the surgery.

## Discussion

This case report highlights the marked attenuation of amplitude of transcranial MEPs after an intravenous bolus administration of ketamine. It is widely believed that ketamine does not affect MEP amplitude [3]. However, in our patient, the amplitude was significantly decreased 3 minutes after administration of ketamine 1.25 mg/kg. Our experience with this patient suggests that ketamine may indeed affect the MEP amplitude, so anesthesiologists need to be cautious when considering the timing and dose of intravenous administration of ketamine.

Anesthesiologists believe that ketamine does not affect MEP amplitude. Indeed, bolus administration of ketamine 0.5 mg/kg during either propofol/remifentanil or N_2_O/sufentanil anesthesia has been reported to have no significant influence on the amplitude of MEPs evoked by transcranial electrical stimulation [6, 7].

However, in our patient, it is possible that bolus administration of ketamine 1.25 mg/kg decreased the amplitude of her MEPs. Bolus administration of ketamine was reported to decrease MEP amplitude in a rabbit model [8]. In another study performed in rabbits, continuous infusion of *S*(+)-ketamine decreased the MEP amplitude in a dose-dependent manner without affecting spinal cord evoked potentials [9]. These findings suggest that ketamine may inhibit the activity of spinal motoneurons. Although Kalkman *et al.* [3] concluded that ketamine 1 mg/kg did not affect MEP amplitude in humans, it was suspected that the amplitude was reduced by administration of ketamine to approximately 50% of control values in two of five study participants. In our patient, although we used a clinically appropriate 1.25 mg/kg dose of ketamine 3 minutes before MEP recordings were started, the waveforms of the MEPs disappeared almost completely. No drugs were administered in addition to ketamine in this patient, and the surgery had not yet started. In addition, her vital signs were stable. Therefore, we hypothesized that a higher effect site concentration of ketamine, which was achieved by bolus intravenous administration, might have affected the MEP amplitude.

Subanesthetic doses of ketamine may improve the quality of postoperative analgesia. Intraoperative and postoperative administration of low-dose ketamine reduced cumulative morphine consumption in patients who had undergone posterior spinal fusion for adolescent idiopathic scoliosis [4]. In a recent meta-analysis, administration of ketamine was reported to decrease both cumulative morphine consumption and the incidence of postoperative nausea and vomiting [10]. At our hospital, anesthesiologists have used ketamine for intraoperative and postoperative analgesia during complex spine surgeries, because these procedures are particularly painful [5] and because it is believed that ketamine does not affect MEP monitoring. If ketamine does affect the MEP amplitude, especially in complex spine surgery where intraoperative neurophysiologic monitoring is required, bolus administration of ketamine would interfere with monitoring. If our hypothesis is true, the anesthesiologist should adjust the timing of administration of ketamine so that the surgeon can appropriately modify the surgical procedure according to the correct results of MEP monitoring. A prospective, randomized clinical trial is needed to confirm whether ketamine truly affects the MEP amplitude.

## Conclusions

This report describes a patient in whom the MEP amplitude was markedly attenuated after intravenous bolus injection of ketamine 1.25 mg/kg. This case suggests that the timing of ketamine administration should be considered carefully, especially when the surgeon is performing a procedure that requires close attention to the results of neurophysiologic monitoring.

## Abbreviations

MEP: Transcranial motor-evoked potential
SSEP: Somatosensory evoked potential
